# Influence of Menstrual Cycle on Internal and External Load in Professional Women Basketball Players

**DOI:** 10.3390/healthcare11060822

**Published:** 2023-03-10

**Authors:** María de los Ángeles Arenas-Pareja, Pablo López-Sierra, Sergio J. Ibáñez, Javier García-Rubio

**Affiliations:** Optimization of Training and Sports Performance Research Group (GOERD), Faculty of Sports Science, Universidad de Extremadura, 10003 Cáceres, Spain; marenasp@alumnos.unex.es (M.d.l.Á.A.-P.);

**Keywords:** menstrual cycle, external load, performance, women’s basketball, basketball

## Abstract

The menstrual cycle can be seen as a potential determinant of performance. This study aims to analyze the influence of the menstrual cycle in women on sports performance, more specifically on the internal and external load of professional women basketball players. The sample consisted of 16 women players and 14 training sessions were recorded. A descriptive analysis of the mean and standard deviation of the variables according to the different phases of the menstrual cycle was performed, as well as an ANCOVA, partial Eta2 effect size criteria, and Bonferroni’s Post Hoc test to identify differences among phases. The results establish that ovulation is the phase in which higher values of external load are recorded and, therefore, the late follicular phase is the time of the cycle where a greater intensity in explosive distance, accelerations and decelerations are recorded. Considering women’s hormonal cycles, understanding their function and the individual characteristics of each athlete is essential since it allows for the development of specific training, the prevention of injuries and therefore positively affects the performance of women players. To this end, individual training profiles should be created in specific contexts, not following general rules. In addition, psychological factors and the specific position of the athletes should be monitored.

## 1. Introduction

The scientific literature on women’s basketball is very limited, and is mainly on performance parameters. The frequent exclusion of women athletes from research studies has been justified, among other reasons, by the hormonal variations of the menstrual cycle (MC). The fluctuations of the hormones during the cycle generate a series of confounding factors which are difficult to control and affect performance, which makes it difficult to design the studies and later interpret the findings [1,2,3]. In the field of research of Sports Sciences, there are more articles that study a population of men and the number of investigations on women athletes is much lower. Specifically, women’s basketball is the sport with the most federative licenses in Spain [4]. Being aware of this, it is considered necessary to delve into the effects that the MC can have on the performance of female basketball players to help coaches better understand this influence and provide information that will allow them to properly adjust the training plan in order to optimize performance results.

The process of optimizing performance in women’s basketball involves respecting principles of sport training such as individualization and specificity [5]. Quantification of the training load is a fundamental tool for obtaining objective, valid and useful information for the coach and the technical team [1]. The workload that the women athletes experience can be divided into: (I) the external load that depends on volume (time and distance), intensity of the actions, duration and density of the efforts and pauses, analysis of the actions (techniques, jumps, passes, impacts, sprints, accelerations, etc.), and volume of the musculature involved; and (II) the internal load, which depends on the heart rate, maximum oxygen consumption, lactate concentration, enzymatic parameters, modification of minerals and ions, hormonal changes or other biochemical variations [6].

Historically, the designs of training programs were carried out with reference to men without respecting the physiological differences that may exist between men and women [7]. The lack of adaptability of the training programs, as they did not take the MC into account in planning and individualization, can have a negative effect on the preparation and sport performances of the women athletes.

**Figure 1:**
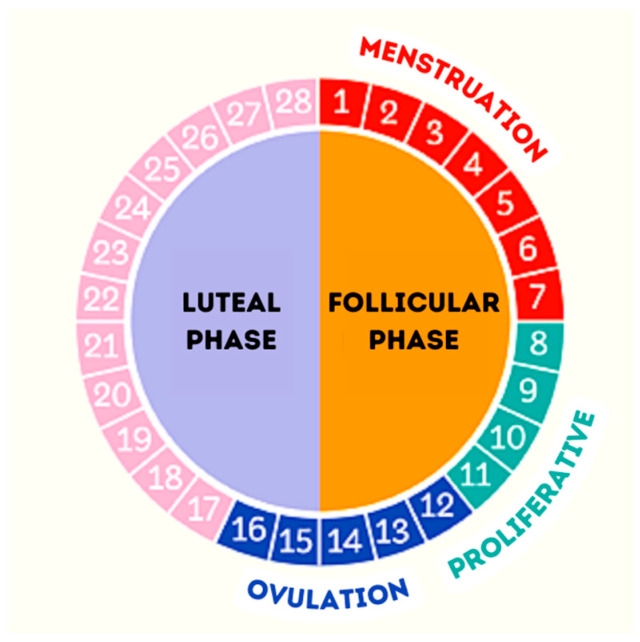
Phases of the MC.

There is considerable interindividual variability among women of the causes of the menstrual disorders (amenorrhea, oligomenorrhea, irregular menstruation, anovulation…) that seems to be more frequent among elite women athletes [8]. The usual duration of the MC in the population is 28 days, with variations of up to seven days [9]. In women athletes, 28-day cycles are observed in 60% of cases, 21-day cycles in 28% and 30–35-day cycles in just 10–12% [10]. Table 1 presents the phases that the MC can be divided into according to the main hormonal changes that are produced [11]. Figure 1 presents the estimated duration of the different phases during the 28 days of the MC [12].

Thanks to technologies such as hormone-specific tests, serial measurements of estrogens, progesterone metabolites, the LH hormone, or new methodologies currently under investigation, it has been possible to improve the monitoring of the woman’s cycle [13,14,15,16,17,18,19]. Today it is possible to integrate the signals, recording, and calculation of fertility signs with the intention of improving the prediction and detection of each phase of the MC. Although the integration of these resources could be interesting to improve the recording approach, an accessible and achievable individualized approach to differentiate each phase using mobile applications is also useful [20,21].

Physical performance changes during the MC due to different mechanisms which provoke alterations in muscular activation, metabolism of substrates, thermoregulation and body composition. The concentration of female hormones could be responsible for the alteration in the production of force and may affect strength and muscle power. Estrogen has a neuroexcitatory effect and progesterone inhibits cortical excitability [22]; these neuroexcitatory and inhibitory effects mean that estrogen and progesterone have a positive and negative relation with the production of strength, respectively [23,24]. The hypothesis is that higher levels of strength and power would be produced when progesterone is maintained low during the follicular phase, especially when the estrogens reach their peak during ovulation, and the lower strength results would be produced in the luteal phase when progesterone is high. In this regard, several studies, using specific strength programs, have shown that there is a greater gain and production of strength in the follicular phase [25,26].

Thus, the MC can be seen as a potential determinant of performance and, in spite of this, it continues to be ignored as hormonal fluctuations are not properly considered in the individualization of women’s training. However, new studies have arisen recently that include women athletes and investigate the adaptations to training according to their MC [27,28].

Many researchers state that women who present a regular MC do not need to adjust the cycle to maximize performance [29]. It is difficult to confirm since all the measurements on which they are based are done in laboratory conditions with isolated tests. These conditions are very different from a real training situation, especially in team sports with an open context, such as basketball, where various factors or processes, such as decision making and the ability to perceive the environment and opponents, can be disrupted by the phases of the cycle, affecting the final performance of the players [30]. It is therefore advisable to examine what happens in a real situation.

As far as we can ascertain, in the current studies of physical performance in women’s basketball, either the MC of the players has not been taken into account or it is taken into account in laboratory conditions. Thus, the purpose of this study was to analyze the effects of the MC on the internal and external load of professional women basketball players in a real training situation.

## 2. Materials and Methods

### 2.1. Research Design

This investigation used an associative strategy which sought to examine the differences among groups [31]. It is included in the framework of observational cross-sectional studies as no type of intervention was conducted. Researchers do not manipulate the intervention process of the participant sample; they only record information about the object of study, giving an ecological treatment to the development of the training sessions. Based on this, it is research that can also be included within ex post facto studies, also called correlational studies [32].

### 2.2. Sample and Population

This research was conducted with two professional women’s teams from the Challenge Women’s League, the second level of women’s basketball in Spain. The participants were 18 professional women basketball players (age: 23 ± 3.1 years; height: 177.4 ± 8.7 cm). The reality of sports training is that the number of players analyzed is always relatively low, since it is a high competition context. This reality of the high-level sports context does not limit the validity of its results [33]. Finally, only 14 female players were included in the study as they were the ones who met the inclusion criteria.

#### Eligibility Criteria

The following criteria were used to select the cases participating in the sample: (i) only female basketball players belonging to the teams analyzed were included in this study; (ii) to be considered a case study, the female player must have participated in at least 80% of the training sessions; (iii) the player must have not been taking a contraceptive pill, at least in the last six months; (iv) the player must have regular duration menstrual cycles; (v) the player must not have suffered injuries in the last four weeks of training.

Exclusion criteria were: (i) participating in less than 80% of the training sessions in the period analyzed; (ii) taking a contraceptive pill or have taken it in the last six months; (iii) have irregular duration menstrual cycles; (iv) have suffered injuries in the last four weeks of training; (v) did not participate voluntarily.

All training sessions of semi-professional women’s basketball teams that agreed to participate voluntarily in the study were recorded. The data presented in this study came from 14 training sessions. The designs of the tasks were elaborated by the coaching staff of each team according to the sports objectives of each team, maintaining the same structure they had been developing since the beginning of the season. The structure of the recorded sessions consisted of two clearly differentiated parts: the first part of warm-up with a duration of 21.47 ± 8.13 min and a main part in which the coaches developed technical-tactical tasks with a duration of 67.52 ± 14.80 min.

### 2.3. Measurements

The independent variable was the phase of the MC, categorized as Menstruation, Proliferative, Ovulation and Luteal. Nineteen dependent variables were recorded (see Table 2) and divided into two groups: Internal Load (IL) and External Load (EL). All took the training session as the covariate.

### 2.4. Instruments

**Figure 2:**
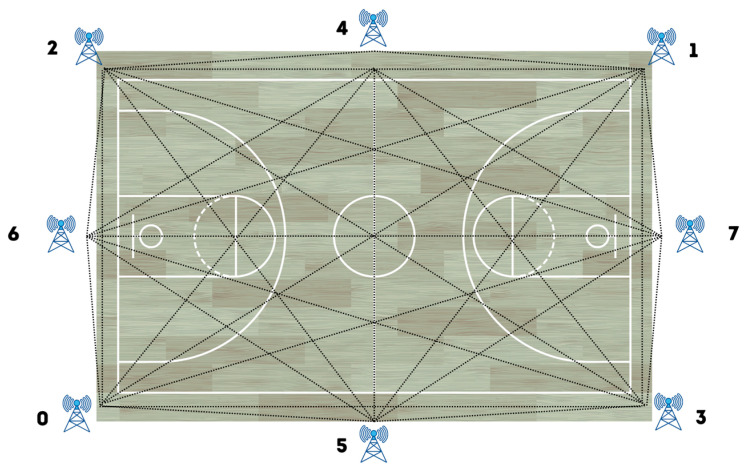
System of UWB antennas on a basketball court.

To record the variables, data were obtained using a WIMUPRO^TM^ (Position-tracking System) (RealTrack Systems, Almería, Spain). Ultra-wideband (UWB) technology was used for positioning data; this records sport information indoors with greater measurement accuracy thanks to the positioning of antennas (Figure 2). The UWB system is adjusted to the reference field by being carried around the perimeter and recognizing it as the reference system [4,34].

To keep track of the MC, each player used the Clue mobile app to record her period derived symptoms [20,35,36].

### 2.5. Procedure

All the players and coaching staff were informed about the protocol to be carried out, as well as had their queries or clarifying doubts answered. Subsequently, all the members signed their informed consent, and the research was carried out following the ethical criteria of the Declaration of Helsinki (2013) and was approved by the Bioethics Committee of the University (233/2019).

Before the data collection, all the players answered a questionnaire [37,38] about their MC to know the situation of each one (phase of the cycle in which they were, duration of the last cycle, use or not of the contraceptive pill, regularity of the cycle, etc.). In each training session we always asked the players what phase of the cycle they were in to keep track of it. All of them always consulted it in the mobile app Clue that each one uses to keep their own records.

**Figure 3:**
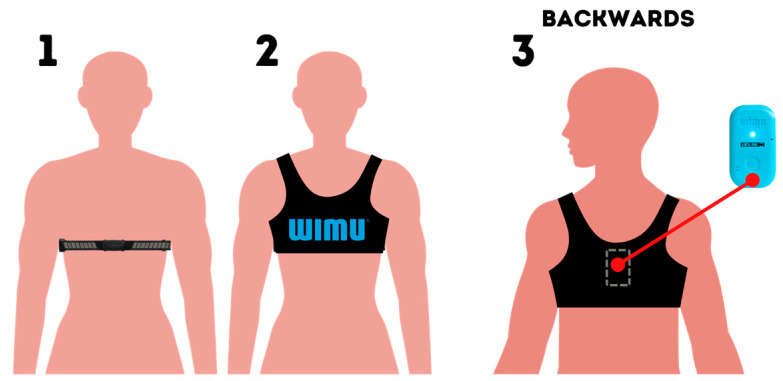
Heart rate band and inertial device placement.

In order to analyze the training session, the UWB system was calibrated about an hour before the start of each session and the WIMUPRO^TM^ inertial devices were synchronized with the UWB system using ANT + technology. Each player was equipped with a GARMIN^TM^ heart rate band and a WIMUPRO^TM^ inertial device placed in a specific anatomic harness fitted on the upper back (Figure 3). This protocol was carried out before the start of each training session after a period for familiarization during the first data collection session [4,34].

### 2.6. Data Analysis

First, a descriptive analysis was made using means and standard deviation, analyzing the variables according to the phases of the MC. Then, an inferential analysis was performed with an ANCOVA, a statistical procedure that makes it possible to eliminate the heterogeneity caused by the variable of interest (dependent variable) due to the influence of one of more quantitative variables (covariable, in this case the training sessions). Moreover, the differences among the phases were identified in more detail with Bonferroni’s Post Hoc test. Effect size was calculated using partial *Eta^2^*, considering the effect size of 0.01–0.06 as small, 0.06–0.14 as medium and >0.14 as large [39]. The statistical analyses were performed with jamovi v2.3.18 [40]. Significance was set at *p* < 0.05.

## 3. Results

Based on the results of the MC questionnaire answered on the first day by the players, the following information is obtained from our sample: participants have an average cycle length of 28 days, with a standard deviation of ±2.8 days.

Table 3 shows the results of the descriptive analysis of the variables of the internal and external loads according to the phases of the MC.

Analyzing the variables relative to distance, accelerations, and decelerations, a greater intensity of external load can be seen in the proliferative and ovulation phases, with the ovulation phase being very significant. Regarding the internal load variables relative to heart rate, they were uniform over the four phases of the MC.

Furthermore, it was established whether there were statistically significant differences or not among the variables examined and the phases of the MC (Table 4).

The results of the ANCOVA show that there exist statistically significant differences among the phases with a medium effect size in maximum accelerations (F = 3.26; *p* = 0.026; η^2^*p* = 0.115), maximum decelerations (F = 3.26; *p* = 0.026; η^2^*p* = 0.115), and high take-offs (F = 3.694; *p* = 0.015; η^2^*p* = 0.127). Specifically, these differences are found between (b) Menstruation-Ovulation in maximum decelerations (*p* = 0.016) and between (c) Menstruation-Luteal in high take-offs (*p* = 0.014). Moreover, we found statistically significant differences among phases with a large effect size in explosive distance (F = 5.25; *p* = 0.002; η^2^*p* = 0.172), mean acceleration (F = 6.43; *p* = <0.001; η^2^*p* = 0.205), mean deceleration (F = 7.00; *p* = <.001; η^2^*p* = 0.219), high accelerations (nº: F = 5.77; *p* = 0.001; η^2^*p* = 0.187; meters: F = 4.97; *p* = 0.003; η^2^*p* = 0.166) and high decelerations (n°: F = 6.18; *p* = <0.001; η^2^*p* = 0.198; meters: F = 4.67; *p* = 0.005; η^2^*p* = 0.157). Specifically, these differences appear between (b) Menstruation-Ovulation in explosive distance (*p* = 0.001), mean acceleration (*p* = <0.001), mean deceleration (*p* = <0.001), high accelerations (n°: *p* = <0.001; meters: *p* = 0.002) and high decelerations (n°: *p* = <0.001; meters: *p* = 0.003) and between (d) Proliferative-Ovulation and (f) Ovulation-Luteal in explosive distance (*p* = 0.021; *p* = 0.006), mean acceleration (*p* = 0.011; *p* = 0.014), mean deceleration (*p* = 0.007; *p* = 0.009), high accelerations (n°: *p* = 0.040; *p* = 0.013; meters (only in f): *p* = 0.0.24).

In addition, it is observed that there are no significant differences with respect to the internal load.

## 4. Discussion

The purpose of this investigation was to discover how the MC affects the external load of professional women basketball players. The results show that the main differences are found in the magnitude of the accelerations and decelerations and the explosive distance covered, with ovulation being the phase in which the players presented higher values of external load and, thus, the late follicular phase is the moment in the cycle when the greatest intensity was recorded in explosive distance, acceleration and decelerations.

The MC has shown that it is a conditioner of performance [41,42]. In fact, the worst results should be found in the late luteal phase and the early follicular phase because there is a variation in the hormones, both progesterone and estrogens [43]. It is probably the decrease in both that is manifested in this dip in performance, meaning that the organism is not prepared to reach its maximum level. In the late follicular phase, the levels of these hormones increase and prepare the body to support high loads. In this regard, previous studies have shown significantly better performance in intermittent endurance (14%) [44] and muscle strength (26%) [24] in the follicular phase compared with the luteal phase.

Basketball is defined as an intermittent sport in which explosive actions stand out [31]. It requires a great capacity to carry out acyclical actions on the part of the players and a rapid speed of execution. These actions include changes in direction, accelerations and decelerations, lateral movements, jumps, contact and specific skills [45].

Taylor et al. [46] found that accelerations represent 5.3% of the distance covered in competition and that women cover greater distances sprinting than men. Different studies report that accelerations, together with jumps, are the type of key effort responsible for scoring in basketball and, thus, the ones on which play performance depends [47,48,49,50]. For this reason, accelerations and decelerations have been used to determine intensity zones during competition [51]. Therefore, it is observed that the acceleration capacity is one of the determining variables of basketball performance and, after analyzing the results obtained in this research, it is also the main variable that is affected by the MC. This is one more reason to take into account the MC when planning the training of female players in order to try to minimally affect this ability and thus achieve the least possible decrease in performance.

In the study by Graja et al. [52], it was observed that during the late follicular phase fatigability was reduced and a lower fatigue index was found together with a higher power peak in the final intervals of a repeated cycle sprint exercise. Furthermore, Cook et al. [53] found that peak power increased during ovulation, also observing that the participants’ motivation for training and competing was also greater in this phase. Pallavi et al. [24] showed a higher fatigue index during menstruation, followed by the luteal phase and the follicular phase, attributing it to the psychological component as bleeding has a negative effect on performance due to preconceived anxiety about it, and that it can even be related to physical performance, as the loss of blood can also affect it.

It is of vital importance to consider women’s hormonal cycles, understand their functioning and the individual characteristics of each woman athlete as it permits prescribing specific training, preventing injuries and, thus, positively influences the athlete’s performance. For example, referring to the weekly frequency of training sessions, a greater frequency at the end of the follicular phase is the most effective option for increasing performance [54]. A good starting point for improving training sessions would be to organize physical conditioning based on the four phases of the MC so that each player can manage their sports activity and govern their body according to their needs [55]. Moreover, it would also be interesting to bear in mind the specific playing position of each player, as it has been shown that it influences the number and intensity of efforts to be made during competition [56].

The negative and positive physical, psychological, or emotional effects depend on the menstrual phase in which each athlete finds herself, which is why the importance of planning training sessions aimed at each particular player is highlighted [57]. The question now is to individualize and carry out individual studies to correct concrete problems in specific contexts, not to establish general rules. In the last few years, some sport institutions have begun to adapt their training programs to the MC [58]. Using daily recording, it is possible to adapt the training session to the moment in the period in which the player finds herself and to know how menstruation affects each one, as, although there are symptoms that are considered generic, not all women have the same experience (different frequencies, more or less intense pain…) [37].

Two limitations of the present study are shown which are linked to future prospects for the improvement of this research: ecological studies have as one of their limitations the small sample size. To improve the generality of the results it would be necessary to increase the sample with other professional female basketball players and track over a longer period. In addition, to improve the reliability of the results, this research could be complemented with the use of biological markers of estrogens, progesterone and the LH hormone. In this study they were not used to show coaching staffs that with scarce resources they can obtain very valid information on how the MC affects their players [59]. For this reason, the results need to be interpreted with care.

## 5. Conclusions

The importance of the MC has been shown in the efforts that the female players may perform. In a sport such as basketball, characterized by explosive efforts, understanding this influence can help to control and establish workloads in female basketball players to generate, at each moment, optimal training conditions.

Based on the findings, it is suggested that the planning of the training of women basketball players be adjusted to their MC. The late follicular phase and ovulation seem to be the best stage for training focused on speed and explosiveness, developing more difficult jumps and sprints, as it is also a hormonal period which favors working on strength and maximum strength. The peaks of maximum performance occur in the ovulation phase, so it would be advisable to program the measurement of the maximum values of the different efforts at that moment. In contrast, in the luteal phase it would be interesting to work on lower intensities than usual because tolerance to fatigue decreases and to carry out training that varies between the minimum effective volume and the adaptative maximum, making these days suitable for focusing on cardiovascular work [43].

Future research should study the role of the MC in sport performance in a real situation of training and competition during a longer period using more reliable methods of recording, such as biological markers, to understand the implications of hormonal variations and even establish individual performance profiles of women basketball players based on their MC. Further research is needed to determine if the fluctuations in motivation and other aspects related to mood, which are altered by the MC and can influence the slowing of motor responses, affect different performance results.

## Figures and Tables

**Table 1:** Phases of the MC and main hormonal variations.

Phase		Hormonal Variations
Follicular	Menstruation	The hormonal levels of estrogens and progesterone are low.
	Proliferative	The levels of estrogens and the luteinizing hormone (LH) begin to increase.
Ovulation		When the levels of estrogens are sufficiently high there is a drastic increase in the levels of LH. This peak causes ovulation (release of the ovule from the ovary).
Luteal		The levels of progesterone reach their maximum approximately in the middle of this phase.

**Table 2:** Study variables.

Variable		Variable Dimensions
IL	Heart rate	Maximum (HRMax): highest bpm. Mean (HRAvg): average bpm.
EL	Distance	Total, covered (D): meters. Explosive (DExpl): meters with an acceleration >1.12 m/s^2^.
	Accelerations	(AC) count: No. of positive speed changes (m/s^2^). Maximum (AC): greatest absolute acceleration. Mean (ACAvg): average of accelerations. High (HighAC): accelerations >3 m/s^2^.
	Decelerations	(DEC) count: No. of negative speed changes (m/s^2^). Maximum (DECMax): greatest absolute deceleration. Mean (DECAvg): average of decelerations. High (HighDEC): decelerations >−3 m/s^2^.
	Speed	Sprints (S): No. of sprints at >21 km/h. Maximum (VMax): greatest speed (km/h).
	Neuromuscular load	Player Load (PL): vectorial sum of accelerations carried out in the three axes (X, Y, Z) provided by the inertial device.
	Jumps	(J) count: No. of elevations from the court with an impulse which implies more than 400 ms of flight time before landing again. High take-offs (HighT): No. of jumps with a take-off force >3 G.

**Table 3:** Results of the descriptive analysis according to the phases of the MC.

	Menstruation	Proliferative	Ovulation	Luteal
	M ± SD	M ± SD	M ± SD	M ± SD
D	4987.89 ± 986.46	5046.70 ± 1121.99	6087.04 ± 469.12	5062.08 ± 1489.43
DExpl	716.41 ± 273.24	924.46 ± 378.23	1426.68 ± 529.50	861.42 ± 418.14
AC	2514.63 ± 568.02	2670.62 ± 865.58	2285.40 ± 1183.92	2454.26 ± 791.82
DEC	2523.16 ± 569.53	2697.10 ± 837.07	2328.00 ± 1183.92	2488.26 ± 741.04
ACMax	6.84 ± 1.79	7.80 ± 1.98	8.33 ± 1.52	8.14 ± 1.63
DECMax	−7.06 ± 1.84	−7.78 ± 1.81	−8.78 ± 0.85	−7.70 ± 1.63
ACAvg	1.12 ± 0.22	1.50 ± 0.57	1.93 ± 0.83	1.48 ± 0.69
DECAvg	−1.08 ± 0.20	−1.42 ± 0.50	−1.82 ± 0.74	−1.39 ± 0.59
HighAC (n)	129.37 ± 113.33	302.90 ± 243.18	447.60 ± 367.41	249.51 ± 256.77
HighDEC (n)	113.95 ± 90.20	267.05 ± 216.91	406.80 ± 322.36	234.11 ± 229.29
HighAC (m)	266.27 ± 180.17	514.32 ± 364.35	759.15 ± 545.42	440.49 ± 381.06
HighDEC (m)	253.72 ± 160.23	476.52 ± 341.31	704.31 ± 521.49	437.29 ± 369.77
HRMax	171.61 ± 14.94	164.20 ± 33.66	177.20 ± 16.48	178.49 ± 14.22
HRAvg	129.67 ± 15.40	126.70 ± 22.18	140.20 ± 15.55	136.27 ± 16.87
S	31.21 ± 18.28	36.62 ± 20.92	48.60 ± 22.51	39.11 ± 21.87
VMax	16.43 ± 2.10	18.64 ± 5.08	20.62 ± 4.23	19.01 ± 6.57
PL	53.92 ± 10.00	52.20 ± 15.55	45.33 ± 20.14	55.01 ± 12.37
J	55.21 ± 24.21	46.15 ± 24.63	36.40 ± 33.58	45.32 ± 32.94
HighT	11.74 ± 4.63	7.80 ± 6.89	6.60 ± 6.31	7.05 ± 3.99

Variables: Total Distance (D); Explosive Distance (DExpl); Accelerations number (AC); Decelerations number (DEC), Maximum Acceleration (ACMax): Maximum Deceleration (DECMax); Mean Acceleration (ACAvg); Mean Deceleration (DECAvg); High Accelerations (HighAC); High Decelerations (HighDEC); Maximum Heart Rate (HRMax); Mean Heart Rate (HRAvg); Sprints (S); Maximum Speed (Vmax); Player Load (PL); Jumps number (J); Jumps High Take-offs (HighT).

**Table 4:** Significant differences among the phases of the MC.

	F	*p*	η^2^*p*
D	0.794	0.501	0.030
DExpl	5.25	0.002 *^,bdf^	0.172
AC	0.347	0.791	0.014
DEC	0.313	0.816	0.012
ACMax	3.26	0.026 *	0.115
DECMax	3.26	0.026 *^,b^	0.115
ACAvg	6.43	<0.001 *^,bdf^	0.205
DECAvg	7.00	<0.001 *^,bdf^	0.219
HighAC (n)	5.77	0.001 *^,bdf^	0.187
HighDEC (n)	6.18	<0.001 *^,bdf^	0.198
HighAC (m)	4.97	0.003 *^,bf^	0.166
HighDEC (m)	4.67	0.005 *^,b^	0.157
HRMax	2.32	0.082	0.085
HRAvg	2.04	0.115	0.076
S	1.078	0.364	0.041
VMax	1.85	0.145	0.069
PL	0.746	0.528	0.028
J	0.783	0.507	0.030
HighT	3.694	0.015 *^c^	0.127

Variables: Total Distance (D); Explosive Distance (DExpl); Accelerations number (AC); Decelerations number (DEC), Maximum Acceleration (ACMax): Maximum Deceleration (DECMax); Mean Acceleration (ACAvg); Mean Deceleration (DECAvg); High Accelerations (HighAC); High Decelerations (HighDEC); Maximum Heart Rate (HRMax); Mean Heart Rate (HRAvg); Sprints (S); Maximum Speed (Vmax); Player Load (PL); Jumps number (J); Jumps High Take-offs (HighT). * *p* < 0.05. Note: statistically significant differences between b: Menstruation-Ovulation; c: Menstruation-Luteal; d: Proliferative-Ovulation; f: Ovulation-Luteal.

## Data Availability

Not applicable.
